# Comparison of three different PCR protocols for the detection of ferlaviruses

**DOI:** 10.1186/s12917-019-2028-0

**Published:** 2019-08-06

**Authors:** Ekaterina Kolesnik, Timothy H. Hyndman, Elisabeth Müller, Michael Pees, Rachel E. Marschang

**Affiliations:** 1 Laboklin GmbH & Co. KG, Steubenstraße 4, 97688 Bad Kissingen, Germany; 2 School of Veterinary Medicine, Murdoch University, Murdoch, Western Australia 6150 Australia; 3 Clinic for Birds and Reptiles, University of Leipzig, An den Tierkliniken 17, 04103 Leipzig, Germany

**Keywords:** Paramyxovirus, Snake, Reptile, Diagnosis, Lung, Genogroup

## Abstract

**Background:**

Ferlaviruses are important pathogens in snakes often associated with respiratory and neurological disease. The detection of ferlaviral RNA by PCR is considered to be the most reliable method for the diagnosis of infection. The PCRs that have been used most commonly for this purpose have not been properly assessed to determine their sensitivity, specificity and ability to detect the known genetic diversity of this group of viruses. The aim of this study was to compare three published PCR protocols so that a single method could be recommended to laboratories that perform this testing.

**Results:**

Comparisons were carried out using cell culture isolates and tissues from snakes infected with specific virus genotypes. A single round PCR targeting a short segment of the viral polymerase (L) gene provided the highest sensitivity and specificity, and detected isolated ferlaviruses from all four described genogroups, as well as from tissues of infected snakes.

**Conclusion:**

A broadly-reactive PCR for the detection of all known ferlaviruses was found to provide a good combination of detection limit, specificity and speed. Based on these criteria, this method is recommended for the diagnosis of ferlavirus infections.

**Electronic supplementary material:**

The online version of this article (10.1186/s12917-019-2028-0) contains supplementary material, which is available to authorized users.

## Background

Paramyxoviruses (PMV) are enveloped RNA viruses belonging to the order *Mononegavirales* and contain a number of important pathogens of mammals as well as both avian and non-avian reptiles [1]. In reptiles, PMVs most often affect snakes [2]. Infections in lizards and chelonians have also been described but seem to be rarer [3–8]. The PMVs that have been genetically characterised in reptiles have so far all belonged to the genus *Ferlavirus*. The name ferlavirus was derived from the Fer-de-Lance viper (*Bothrops atrox*), as this was the reported host species that the first ferlaviruses were isolated from during an outbreak of respiratory disease in a Swiss serpentarium in 1972 [9, 10]. Since this initial discovery, ferlavirus infections in snakes have been regularly implicated in disease outbreaks [11–16]. Clinical signs can be variable and non-specific, they most often involve the respiratory system, but neurological disease has also been reported in several cases [2]. Four different strains of ferlaviruses have been described based on partial polymerase (L), hemagglutinin (HN) und unknown (U) gene sequence analysis. These are currently referred to as genogroups A, B, C, and “tortoise” [16–19] although formal classification has not yet been ratified by the International Committee on Taxonomy of Viruses (ICTV). Detection of ferlaviruses in clinical samples, such as lung washes, swabs and tissue samples, has been achieved by virus isolation in cell culture and by reverse transcriptase PCR for detection of viral RNA. Several different PCRs have been described. A nested PCR targeting a portion of the L gene was first described by Ahne et al. [17]. The L gene is a well conserved gene among PMVs [19, 20] and this PCR has been used to detect all of the reptile ferlavirus genotypes described so far [19]. There is frequent use of this PCR in published work [15, 16, 21–23] and also by diagnostic laboratories [24]. However, one study describing ferlavirus detection in 19 of 495 clinical samples from reptiles showed that 8.82% of negative samples produced PCR products that were of a similar size (500–600 bp) to those expected of a true positive [25]. A direct comparison between PCR, using the method described by Ahne et al. [17], and virus isolation using viper heart cells (VH2) was carried out by Pees et al. [21]. In this study, 21 cases of ferlaviral infection were detected by virus isolation only. However; overall, more infections were detected by PCR than by virus isolation. Tong et al. [26] developed and described a number of broadly-reactive semi-nested PCRs for the detection of a segment of the L gene of a wide range of paramyxoviruses and pneumoviruses. Testing for ferlavirus could be achieved using a PCR designed to detect all known paramyxoviruses (pan-*Paramyxoviridae* [*Paramyxovirinae* using the taxonomy accepted at the time]) or one designed to more specifically detect the clades of paramyxovirus that include the henipaviruses, morbilliviruses, respiroviruses and the ferlaviruses. The suitability of these two PCRs by Tong et al. [26] to detect the known ferlavirus genogroups has not been previously examined. The detection of ferlaviral RNA by PCR was also described by Hyndman et al. [27] as part of an investigation of another snake mononegavirus named sunshinevirus.

The aim of our study was to compare three of the L-gene PCRs described in the studies by Ahne et al. [17], Tong et al. [26] and Hyndman et al. [27] based on their respective detection limits, specificity and ability to detect all known ferlavirus genotypes.

## Results

The measured concentrations of RNA extracted from the cell culture isolates and the results of the dilution series are listed in Table 1. PCR I [17] and PCR III [27] detected RNA extracted from cell culture isolates of all of the four ferlaviruses included in this study. In contrast, PCR II [26] only detected RNA from the type A and “tortoise” isolates. The detection limits of PCR I and PCR III were similar. Both PCRs detected dilutions of RNA from virus-infected cultures that contained between 5 × 10^− 1^ ng/μL and 5 × 10^− 3^ ng/μL depending on the virus isolate (Table 1), while 5 or 7 ng/μL were necessary for detection by PCR II. All positive PCR products from cell culture isolates were confirmed by sequencing.

**Table 1:** Dilution series of the cell culture isolates tested using three different PCR protocols

Concentration of RNA from cell culture isolates	PCR I [17]	PCR II [26]	PCR III [27]
Xeno-USA99			
5 ng/μL	+	(+)	+
5 × 10^− 1^ ng/μL	+	–	+
5 × 10^− 2^ ng/μL	–	–	((+))
5 × 10^− 3^ ng/μL	–	–	–
5 × 10^− 4^ ng/μL	–	–	–
Crot-GER03			
25 ng/μL	+	–	+
6 ng/μL	+	–	+
5 × 10^− 1^ ng/μL	+	–	+
5 × 10^− 2^ ng/μL	+	–	+
5 × 10^− 3^ ng/μL	+	–	(+)
5 × 10^− 4^ ng/μL	–	–	–
PanGut-GER09			
5 ng/μL	+	–	+
5 × 10^− 1^ ng/μL	+	–	(+)
5 × 10^− 2^ ng/μL	–	–	–
5 × 10^− 3^ ng/μL	–	–	–
5 × 10^− 4^ ng/μL	–	–	–
Ther-GER99			
7 ng/μL	+_+_	+	+
5 × 10^−1^ ng/μL	−_+_	–	+
5 × 10^− 2^ ng/μL	–	–	(+)
5 × 10^− 3^ ng/μL	–	–	–
5 × 10^− 4^ ng/μL	–	–	–

-: negative, +: positive amplification product with a strong band, (+): positive amplification product with a weaker band, ((+)): positive amplification product with a weak band, +_+_: positive amplification product of the expected size, with an unspecific lower band, −_+:_ negative PCR result, with an unspecific lower band

Table 2 shows the results of the three different PCRs for the detection of RNA prepared from tissues of ferlavirus-infected corn snakes. Eleven of the 24 samples from the group infected with a genogroup A isolate were positive with PCR I, one sample was positive with PCR II and 14 samples were positive with PCR III. In the group infected with a genogroup B virus, 14 of 24 samples were positive with PCR I, eight samples were positive with PCR II and 17 samples were positive with PCR III. In the group infected with a genogroup C isolate, 12 of 15 were positive with PCR I, four were positive with PCR II, and 10 were positive with PCR III. A total of 44 tissues tested positive by at least one method. Using these as true positives, the relative sensitivities of the PCRs were: 84.1% for PCR I, 29.5% for PCR II, and 93.2% for PCR III. Differences noted in the strength of the bands on electrophoresis are demonstrated in Additional file 1: Figure S1.

**Table 2:** Tissue samples from experimentally infected corn snakes tested using three different PCR protocols (numbering the same as in Pees et al. [21])

No.	Sample material	PCR I [17]	PCR II [26]	PCR III [27]
Genogroup A				
1	lung	–	–	+
	brain	–	–	–
2	lung	+	(((+)))	+
	brain	–	–	–
3	lung	+	–	+
	brain	–	–	–
4	lung	–	–	+
	brain	–	–	–
5	lung	+	–	+
	brain	+	–	(+)
6	lung	+	–	+
	brain	(+)	–	((+))
7	lung	–	–	–
	brain	–	–	–
8	lung	+	–	+
	brain	+	–	+
9	lung	–	–	+
	brain	+	–	+
10	lung	–	–	–
	brain	–	–	–
11	lung	+	–	+
	brain	+	–	+
12	lung	–	–	–
	brain	–	–	–
Total positive	*n* = 24	11	1	14
Genogroup B				
1	lung	–	((+))	–
	brain	–	–	–
2	lung	+	((+))	+
	brain	–	–	–
3	lung	(+)	–	+
	brain	–	–	–
4	lung	+	(((+)))	+
	brain	–	–	(+)
5	lung	–	((+))	+
	brain	–	–	–
6	lung	(+)	(((+)))	+
	brain	–	–	–
7	lung	+	(((+)))	+
	brain	+	–	+
8	lung	(+)	–	+
	brain	+	–	(+)
9	lung	+	–	+
	brain	+	–	+
10	lung	+	–	+
	brain	+	–	+
11	lung	–	((+))	+
	brain	–	–	–
12	lung	+	((+))	+
	brain	+	–	(+)
Total positive	*n* = 24	14	8	17
Genogroup C				
1	lung	+	–	+
2	lung	+	(+)	+
	brain	+	–	+
3	lung	+	–	((+))
4	lung	+	–	–
	brain	+	–	+
5	lung	+	+	+
6	lung	+	–	(+)
7	lung	+	(+)	+
	brain	–	–	–
8	lung	–	–	–
9	lung	+	–	–
10	lung	+	–	+
11	lung	+	+	+
12	lung	–	–	–
Total positive	*n* = 15	12	4	10

-: negative, +: positive amplification product with a strong band, (+): positive amplification product with a weaker band, ((+)): positive amplification product with a weak band, (((+))): positive amplification product with a very weak band. Examples for different bank strengths are shown in Additional file 1: Figure S1

The statistical evaluation of the results for all genogroups showed highly significant agreement between PCRs I and III (*p* ≤ 0.001, κ = 0.732). Significant agreement was also found between PCR II and III, though to a much lower degree (*p* = 0.024, κ = 0.191). No agreement was found between PCRs I and II. Similar results were found for agreement between results for each genogroup separately, with highly significant agreement between PCRs I and III for genogroups A (*p* ≤ 0.001, κ = 0.752), and B (*p* ≤ 0.001, κ = 0.731), and significant agreement for PCRs I and III for genogroup C (*p* = 0.022, κ = 0.667). No significant agreement was found for PCRs I and II or for PCRs II and III for any of the individual genogroups. With PCR I, non-specific amplicons different in length to the ferlaviral amplicons were detected when testing RNA from the tissue samples; however, these bands were consistently weaker than bands of the correct size (data not shown). RNA from a canine distemper virus tested positive with PCR II, but both of the other PCRs were negative for this virus. RNA from Newcastle disease virus tested negative with all three PCRs. No amplification products were obtained when RNA or DNA from the other, non-paramyxovirus pathogens, were used as templates in any of the three PCRs.

## Discussion

Diagnostic testing for infectious diseases is an important field that must continuously adapt to developing knowledge of pathogens. In reptiles, multiple pathogens have been discovered and understanding of their dissemination and the importance of emerging diseases requires optimization of diagnostic protocols. Several infectious agents have been shown to be important emerging diseases in wild and/or captive reptiles, e.g. *Ophidiomyces ophiodiicola* [28, 29] as well as newly described viruses in the order Nidovirales [30–32], and development and wide-spread use of diagnostic testing continues to expand our knowledge of the importance of individual pathogens.

Ferlaviruses have been known to be important pathogens in reptiles, especially in snakes, since the 1970’s [9, 10], and knowledge about the genetic diversity of these viruses has accumulated since the first description of the full genome of a ferlavirus [16, 19, 20, 33]. The most commonly used method for ferlavirus detection in reptiles has been the nested PCR described by Ahne et al. [17] (PCR I in this study). Although this PCR has been used to detect all known ferlavirus genogroups in reptiles [16, 19, 33] and has been shown to have good analytical sensitivity [21], it has been described as nonspecific, so that sequencing of all PCR products is required [25]. Attempts to optimise this nested PCR did not result in a higher specificity [25]. In an experimental infection study with corn snakes, a combination of isolation in cell culture and PCR showed the highest sensitivity, especially when testing lung samples from dead snakes [21]. However, the combination of PCR with cell culture is not practicable for routine diagnostics. This made it necessary to establish a PCR with an equal or better detection limit and a higher specificity that is able to detect the known genetic variants of ferlaviruses as a standard diagnostic tool.

All of the PCRs tested in this study target portions of the ferlaviral L gene, which encodes the viral RNA-dependent RNA polymerase. This has been shown to be relatively conserved among ferlaviruses and between ferlaviruses and other PMVs [19, 20]. It is the most conserved region of the ferlavirus genome for which PCRs have been previously described. PCR protocols that have targeted other portions of the genome, including the F, HN, and U genes, have not been able to amplify RNA from some described ferlaviruses [17–19].

In addition to PCR I, a broadly reactive PCR targeting the paramyxoviruses most closely related to the ferlaviruses (the henipaviruses, morbilliviruses, and respiroviruses) (PCR II in this study) was chosen since this has previously been used to detect Fer-de-Lance virus, the type virus of the *Ferlavirus* genus [26] and was expected to be able to detect all genetic variants within this genus. We did not assess the pan-*Paramyxoviridae* [pan-Paramyxovirinae] primer set described by Tong et al. [26] as we were unable to detect ferlaviral RNA from any genogroup with it.

PCR III was designed from a variety of partial ferlaviral L-gene sequences, although it had not previously been tested on all ferlavirus genotypes [27].

Since the tissues used in this study were collected at different time points post infection and the RNA prepared from each had been stored for different amounts of time before this study was done, not all of the samples from the experimentally infected corn snakes were expected to be virus positive. The highest percentage of ferlavirus positive samples was detected using PCR III (93.2%), followed by PCR I (84.1%) and finally PCR II (29.5%).

PCR II detected all four ferlavirus genotypes in the corn snake tissues but only detected genogroups A and “tortoise” from cell culture. Ferlaviruses from genogroups A, B and C have been described in different reptiles, including snakes, lizards and tortoises [7, 16, 19, 28]. An experimental transmission study with corn snakes demonstrated differences in pathogenicity between different isolates, with the genogroup B virus used in the study the most pathogenic while the genogroup A isolate was least pathogenic [21]. There are no published studies on the prevalence of the different genogroups in snakes, but in the lab of one of the authors (REM), genogroups A and B appear to be most common, while genogroups C and “tortoise” have only been found in individual cases [16, 19, 33]. This shows that it is important to have a reliable PCR which is able to detect all known ferlavirus genotypes.

In all cases where PCR II detected ferlaviral RNA, the amplicons were weak following gel electrophoresis (data not shown); an observation supported by its detection limit that was inferior to PCRs I and III. PCR II also required the use of a thermocycler with rapid warm-up and cool-down phases (data not shown). For these reasons, PCR II cannot be considered suitable for the detection of ferlaviruses for either diagnostic or research purposes.

PCR I detected a high proportion of ferlavirus positive samples in this study (84.1%). This PCR from Ahne et al. [17] has been described in numerous publications as a suitable diagnostic test for the detection of ferlaviruses (reviewed in [24]). In this study, and in the labs of two of the authors (REM and THH), this PCR has been reasonably non-specific and sequencing is therefore a necessity to distinguish true positives from false positives. In agreement with our findings, a study by Abbas [25] found the specificity of this PCR to be relatively low and false-positive results were common. This PCR has also been reported to cross-react with adenoviruses in some cases [25], although we did not find this with the adenovirus DNA we tested.

The highest number of positive samples were identified by PCR III (93.2%). An advantage of this PCR is that it is a single round PCR, in contrast to both of the other PCRs used in this study. This lowers the risk of contamination and mistakes during processing. It also lowers the costs associated with testing and decreases the turn-around time for the assays. Furthermore, no amplification products of unexpected sizes were observed with this PCR. This is also the only PCR for which the results correlated significantly with both of the other two PCRs tested. Testing with RNA prepared from other reptile pathogens as well as tissues from uninfected snakes showed that PCR III was specific for ferlaviruses, an important prerequisite for its use in diagnostic testing. The amplicon obtained using this PCR is 149 bp, which is relatively short. This is a disadvantage where long segments of ferlaviral nucleic acid are needed for phylogenetic studies, but for diagnostic samples that are often of sub-optimal quality, targeting a shorter segment of the genome is an advantage.

## Conclusion

This study shows that PCR III described by Hyndman et al. [27] is broadly-reactive and provides a good combination of detection limit, specificity and speed. We therefore recommend the use of this PCR for the detection of ferlaviruses in diagnostic samples.

### Methods

The three PCRs that were compared are listed in Table 3. The PCRs described by Ahne et al. [17], Tong et al. [26] and Hyndman et al. [27] are referred to as PCRs I, II and III, respectively, throughout the text.

**Table 3:** Primers used for the three different PCRs

RT-PCR	Primer name	Primer sequence	Expected size of final PCR product (with primers)	Reference
I	F5-outer	5′-GCAGAGATTTTCTCTTTCTT-3’	566 bp	[17]
	R6-outer	5′-AGCTCTCATTTTGTATGTCAT-3’		
	F7-inner	5′-TAGAGGCTGTTACTGCTGC-3’		
	R8-inner	5′-CATCTTGGCAAATAATCAGCC-3’		
II	RES-MOR-HEN-F1	5′-TCITTCTTTAGAACITTYGGNCAYCC-3’	500 bp	[26]
	RES-MOR-HEN-F2	5′-GCCATATTTTGTGGAATAATHATHAAYGG-3’		
	RES-MOR-HEN-R	5′-CTCATTTTGTAIGTCATYTTNGCRAA-3’		
III	S2	5′-GTTATGGCAAATCATGCTGCGATACCTTA-3’	149 bp	[27]
	AS2	5′-CTGATGGGAGATAATGCCTTGTCCTTCAT-3’		

To compare the detection limits of these PCRs, serial dilutions of ferlaviral RNA were tested. Four different ferlaviruses representing each of the known genogroups were used. Ferlaviruses were propagated for this study using VH2 cells (ATCC CCL-140) grown at 28 °C (Table 4). Virus was grown in cells until a cytopathic effect (syncytial cell formation and cell lysis) was seen in approximately 50% of the cells and then frozen at − 20 °C. The frozen cells were then thawed and cells and medium were aliquoted and stored at − 80 °C until further testing. Viral RNA was extracted from 200 μL of frozen-thawed cell culture homogenate using the Roche MagNA Pure 96 System with the MagNA Pure 96 DNA and Viral NA Small Volume Kit (Roche, Mannheim, Germany) according to the manufacturer’s instructions. Quantification of extracted RNA from cell culture was performed by spectrophotometry using a Nanodrop 2000 spectrophotometer (Thermo Fisher Scientific, Germany).

**Table 4:** Ferlavirus isolates used in this study

Virus isolate (GenBank accession no.)	Host species (scientific name)	Genogroup	Reference
Xeno-USA99 (GQ277614)	Flathead knob-scaled lizard (*Xenosaurus platyceps*)	A	[19]
Crot-GER03 (GQ277611)	Timber rattlesnake (*Crotalus horridus*)	B	[19]
PanGut-GER09 (HQ148084)	Eastern corn snake (*Pantherophis guttatus*)	C	[16]
Ther-GER99 (GQ227615)	Hermann’s tortoise (*Testudo hermanni*)	tortoise	[19]

To assess the capacity of these three PCRs to detect the known genetic diversity of ferlaviruses, tissue samples from corn snakes (*Pantherophis guttatus*) experimentally infected with the genotype A, B, or C viruses [21] were tested (Table 2). The snakes were originally acquired from a commercial company and originated from unremarkable collections. Details on the ages and origins of the animals were not known. All animals were euthanized followed by necropsy during or at the end of the infection study. This study met the local as well as international guiding principles for biomedical research, and was approved by the local authority (animal trial no. TVV 61/13). Approximately 5 μg of each tissue sample were placed in MagNA Lyser Green Beads (Roche, Mannheim, Germany) with 500 μL Magna Pure tissue lysis buffer (Roche, Mannheim, Germany) and 50 μL proteinase K (Roth, Karlsruhe, Germany) and homogenized with a MagnaLyser (Roche, Mannheim, Germany) for 40 s at 6500 rpm and then incubated for 1 h at 65 °C. RNA from the homogenized tissue was extracted using the same kit as for cell culture. RNA was stored at − 20 °C until further testing.

RNA extracted from cognate tissues from uninfected snakes, or HPLC water, was used as negative controls. Ferlaviral RNA from cell culture isolates was used as a positive control.

The PCR reaction mix for PCR I [17] was prepared with the RealTime Ready RNA Virus Master-Kit (Roche) according to the manufacturer’s instructions. For the first round, 1 μL of each primer (10 μmol), primers F5-outer and R6-outer, and 5 μL of RNA extract were used in a total volume of 20 μL. Cycling conditions were 50 °C for 15 min, 95 °C for 7 min, 9 touch down cycles at 95 °C for 10 s, 57.5 °C for 30 s (− 1 °C per cycle), and 72 °C for 50 s, and 26 cycles at 95 °C for 10 s, 49.5 °C for 30 s, and 72 °C for 50 s, and 72 °C for 7 min for the final extension step. The second round reaction mix was prepared with the FastStart Essential DNA Probes Master-Kit (Roche), 1 μL of the primers F7-inner and R8-inner (10 μmol each), and 5 μL of the amplification product of the first round in a total volume of 20 μL. Cycling conditions were 95 °C for 15 min, 9 touch down cycles at 95 °C for 45 s, 57.5 °C for 45 s (− 1 °C per cycle), and 72 °C for 45 s, and 30 cycles at 95 °C for 45 s, 49.5 °C for 45 s, and 72 °C for 45 s, and finally 72 °C for 10 min.

For PCR II [26], the reaction mix for the first round was prepared with the RealTime Ready RNA Virus Master-Kit (Roche) according to the manufacturer’s instructions and the primers RES-MOR-HEN-F1 and RES-MOR-HEN-R with the same volumes and concentrations as for the first round for PCR I. Cycling conditions were 60 °C for 1 min, 47 °C for 30 min, 94 ° for 2 min, and than 40 cycles at 94 °C for 15 s, 49 °C for 30 s, 72 °C for 30s and a final extension step at 72 °C for 7 min. The second round of the semi-nested PCR was initially carried out with the Roche FastStart Essential DNA Probes Master Kit as for PCR I, but all samples were negative. For this reason, the reaction mix was prepared with Taq-DNA-Polymerase-Kit (Qiagen) according to the manufacturer’s instructions, including 1 μL of each primer, RES-MOR-HEN-F2 and RES-MOR-HEN-R (10 μmol each), 5 μL of Qiagen buffer, 5 μL of MgCl_2_, 5 μL of dNTPs (2 μmol), 0,5 μL Qiagen-Taq, 27,5 μL HPLC water, and 5 μL of the amplification product of the first round in a total volume of 50 μL. Cycling conditions for the second round were 94 °C for 2 min, 40 cycles at 94 °C for 15 s, 49 °C for 30 s, 72 °C for 30s and 72 °C for 7 min.

PCR III [27] was prepared with the RealTime Ready RNA Virus Master-Kit (Roche) according to the manufacturer’s instructions. The volumes of primers S2 and AS2, and RNA extraction, were the same as for the first rounds of PCR I and II. Cycling conditions were 50 °C for 15 min, 95 °C for 7 min, 9 touch down cycles at 95 °C for 10 s, 57.5 °C for 30 s (− 1 °C per cycle), and 72 °C for 50 s, and 26 cycles at 95 °C for 10 s, 49.5 °C for 30 s, and 72 °C for 50 s, and 72 °C for 7 min for the final extension step.

After amplification, PCR products for all three protocols were visualized by gel electrophoresis. PCR products obtained were purified using a MinElute purification kit (Qiagen, Venlo, Netherlands) according to the manufacturer’s instructions. Sequencing was perfomed using the Big-Dye Terminator v3.1 Cycle Sequencing Kit (Life Technologies, Carlsbad, California) and analyzed on an ABI 3130 sequencer (Applied Bio system by Life Technologies). After removal of primer sequences, the ferlaviral sequences were compared to data in GenBank (National Center for Bio technology Information, Bethesda, Maryland, 20894 USA) online using BLAST (https://blast.ncbi.nlm.nih.gov/Blast.cgi? PAGE_TYPE = BlastSearch).

Specificity was assessed by testing all three PCRs with RNA or DNA from a variety of pathogens that are common in reptiles. RNA and DNA from positive samples or cell culture isolates of several pathogens were used (Table 5). The identity of each was previously confirmed by PCR and sequencing. Furthermore, RNA from canine distemper virus and Newcastle disease virus were also tested with all three PCRs to determine whether the PCRs were able to detect more distantly related paramyxoviruses.

**Table 5:** Pathogens used for specificity testing

Pathogen familiy	Host	Genus or species	Sample type
*Sunviridae*	Python	*Sunshinevirus*	Cell culture isolate
*Reoviridae*	Python	*Orthoreovirus*	Cell culture isolate
*Arenaviridae*	Python	*Reptarenavirus*	Tissue
*Tobaniviridae* (Order *Nidovirales*)	Python	*Pregotovirus* (Subfamily *Serpentovirinae*)	Oral swab
*Herpesviridae*	Tortoise	*Scutavirus* (testudinid herpesvirus 1)	Cell culture isolate
*Herpesviridae*	Tortoise	*Scutavirus* (testudinid herpesvirus 3)	Cell culture isolate
*Adenoviridae*	Python	*Atadenovirus*	Cell culture isolate
*Iridoviridae*	Tortoise	*Ranavirus* (*Common midwife toad virus*-like)	Cell culture isolate
*Picornaviridae*	Tortoise	*Torchivirus*	Cell culture isolate
Mycoplasmataceae	Tortoise	*Mycoplasma* spp.	Nasal wash
Cryptosporidiidae	Lizard	*Cryptosporidium varanii*	Feces
Chlamydiae	Bird	*Chlamydia psittaci*	Feces

Statistical evaluation of the PCR results was conducted using the software SPSS 25.0 (IBM, Armonk, USA). All positives (regardless of the strength of the band) were counted as positive for the evaluation. Since the data acquired were ordinal, non parametric tests were chosen for comparison of the results. The Cohen’s kappa coefficient with exact *p*-value calculation was used to evaluate correlations between the PCR protocols for all genogroups. In case of a significance, as post hoc tests the genogroups were then tested individually, to check for statistical significances between the three protocols used. Significance was assumed with *p* ≤ 0.05, and a high significance level with *p* ≤ 0.001. As the study intention was explorative, no adjustment for multiple comparisons was performed.

## Additional file

Additional file 1: **Figure S1.** Gel electropherogram of PCR products from PCR III according to Hyndman et al. (2012) [27]. Lanes 5, 8, 12, and 13 represent clear positive results, lanes 1 and 4 represent weak positives, while lane 6 represents a very weak positive result. Lane 16 is the positive control, and L is the ladder (sizes shown on right). (DOCX 104 kb)

## Data Availability

Data from this study are available from the corresponding author upon request.

## Abbreviations

F: Fusion
HN: hemagglutinin
HPLC: High-performance liquid chromatography
ICTV: International Committee on Taxonomy of Viruses
L: Polymerase
PCR: Polymerase chain reaction
PCR I: Described by Ahne et al. [17]
PCR II: Described by Tong et al. [26]
PCR III: Described by Hyndman et al. [27]
PMV: Paramyxovirus
RNA: Ribonucleic acid
U: Unknown
VH2: Viper heart cells
