# Supplementary material for: Comparison of three different PCR protocols for the detection of ferlaviruses
Source: BMC Vet Res. 2019 Aug 6;15:281. doi: 10.1186/s12917-019-2028-0 (PMC6685236; doi:10.1186/s12917-019-2028-0)
Supplement: Supplementary file 1 — Figure S1. Gel electropherogram of PCR products from PCR III according to Hyndman et al. (2012) [27]. Lanes 5, 8, 12, and 13 represent clear positive results, lanes 1 and 4 represent weak positives, while lane 6 represents a very weak positive result. Lane 16 is the positive control, and L is the ladder (sizes shown on right). (DOCX 104 kb) [file 12917_2019_2028_MOESM1_ESM.docx]

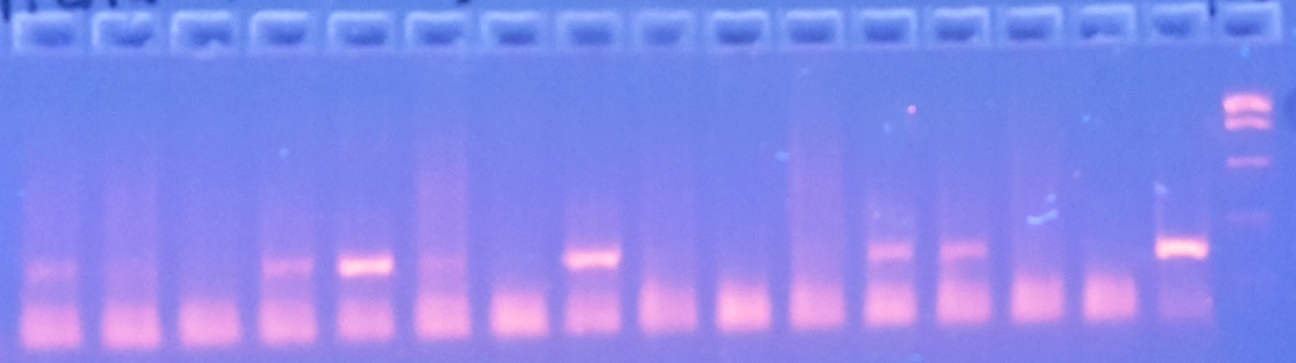


-1000 bp

-800 bp

-400 bp

-200 bp

Additionl file 1: Figure S1 Gel electropherogram of PCR products from PCR III according to Hyndman et al. (2012). Lanes 5, 8, 12, and 13 represent clear positive results, lanes 1 and 4 represent weak positives, while lane 6 represents a very weak positive result. Lane 16 is the positive control, and L is the ladder (sizes shown on right).

| 1 | 2 | 3 | 4 | 5 | 6 | 7 | 8 | 9 | 10 | 11 | 12 | 13 | 14 | 15 | 16 | L |
| --- | --- | --- | --- | --- | --- | --- | --- | --- | --- | --- | --- | --- | --- | --- | --- | --- |
